# Effects of chronic bubble tea administration on behavior and cognition in C57BL/6 mice

**DOI:** 10.3389/fpsyt.2022.1044052

**Published:** 2022-12-07

**Authors:** Yitan Yao, Shengya Shi, Yating Yang, Bei Luo, Mengdie Li, Ling Zhang, Xiaoping Yuan, Huanzhong Liu, Kai Zhang

**Affiliations:** ^1^Department of Psychiatry, Chaohu Hospital of Anhui Medical University, Hefei, China; ^2^Anhui Psychiatric Center, Anhui Medical University, Hefei, China

**Keywords:** bubble tea, sugar-sweetened beverages, metabolic diseases, obesity, antidepressant, cognitive function

## Abstract

**Purpose:**

There is a lack of rigorous experimental evidence to verify the effects of bubble tea on body weight and mental health, especially whether it is an addictive thing.

**Materials and methods:**

Male adult C57BL/6 mice were randomly assigned to two groups, including the bubble-tea and the control group. The change in their body weight was calculated. Behavior tests include novel object recognition test (NORT), elevated plus maze test (EPMT), forced swim test (FST), tail suspension test (TST), conditioned place preference (CPP).

**Results:**

There was a significant time effect on weight change of the two groups (*F* = 36.83, *P* < 0.01). The bubble tea-treated mice spent significantly less time in the open arms, indicating an increase in anxiety (*t* = 2.39, *P* = 0.03). In FST, bubble tea treatment produced a significantly increased immobility time (186.58 ± 29.67 s) as compared to pure water treated group (112.50 ± 46.67 s) (*t* = −3.79, *P* < 0.01). Similarly, the immobility time in the TST was also significantly increased by bubble tea treatment (437.63 ± 27.72 s) compared to the treatment with pure water (340.24 ± 77.22 s) (*t* = −3.36, *P* < 0.01). We investigated the rewarding effects of bubble tea, using the CPP paradigm, which measures the rewarding properties of abused drugs. Independent-samples *t*-test revealed no significant difference between the two groups (*t* = −0.47, *P* = 0.65).

**Conclusion:**

In conclusion, we showed that long-term administration of bubble tea could not induce addictive behavior in mice. Meanwhile, the long-term effects of bubble tea on weight were also very limited. However, long-term consumption of bubble tea can lead to anxiety and depression-like behaviors and impair cognitive function in mice.

## Introduction

Bubble tea, also known as Boba milk tea, is a kind of tea-based beverage, prepared with tea, milk, and other ingredients (1). Different kinds of bubble tea have different ingredients, including chewy tapioca starch balls, fruit jellies, cassava flour, matcha powder, cinnamon powder, cheese, taro, red beans, and black sugar. It was invented in Taiwan in the 1980s and later spread around the world, especially in Mainland China. Now, bubble tea is the most popular beverage among children and adolescents, and young adults in China (2). According to our previous social media survey, some young people drink a cup of bubble tea per day or at least three cups per week. One of the most common types of bubble tea is the ready-made 5-min sweet bubble tea.

Against the backdrop of this widespread popularity, there was also growing concern about the health effects of long-term bubble tea consumption. Some previous studies suggested that long-term administration of bubble tea could lead to overweight among children and adolescents (3, 4). A study also showed the potential of bubble tea to increase body weight and exacerbate type 2 diabetes, metabolic diseases, and several other obesity-related diseases (5).

In addition to body weight, bubble tea was also found to be associated with poor mental status in several studies. Wu and colleagues published the first observational cross-sectional study on the association between bubble tea consumption and anxiety or depressive symptoms among Chinese young adults (2). They found that bubble tea consumption was associated with an increased risk of depression and anxiety in Chinese young adults.

Bubble tea is one kind of sugar-sweetened beverages (SSBs), and whether it causes addiction will be a big issue which should be taken seriously. A high level of sugar consumption has been found to be harmful to mental health in several cross-sectional studies (6–8). One study suggested that excessive sugar intake was a risk factor for addiction after adjustment for other covariables (9). Kumar’s study showed that sucrose overeating augments depressive and anxiety behavior during diabetes (10). Different with clinical study, they used tail suspension test (TST), forced swim test (FST), and elevated plus maze test (EPMT) to measure mice’s depressive and anxiety behavior.

Generally, there is a lack of rigorous experimental evidence to verify the effects of bubble tea on body weight and mental health, especially whether it is an addictive thing. This preclinical study addresses the following research questions (1) Is chronic bubble tea administration an induced addictive behavior? (2) Does routine consumption of bubble tea cause weight gain? (3) Does regular use of bubble tea cause symptoms of anxiety or depression? and (4) Does chronic bubble tea administration have any effect on cognitive function?

## Materials and methods

### Animals

Male adult C57BL/6 mice, aged 8 weeks (body weight 20–25 g), were obtained from the Animal Research Center of Anhui Medical University. Four per cage were housed at a controlled temperature (23 ± 2°C) and under a 12 h light/dark cycle (lights on between 7:00 and 19:00), with *ad libitum* food and water. The first 7 days of the animals’ arrival are used for their habituation to the laboratory environment. After the start of the experimental protocol (first administration of bubble tea), mice were housed individually to monitor the daily water intake. All behavioral test were performed in the first half of their light cycle (07:00–12:00). All the experimental procedures were approved and performed in accordance with the guidelines set out by the Institutional Animal Ethics Committee of Chaohu Hospital of Anhui Medical University (No. 2022-kyxm-05).

### Reagents

Ulemei original milk tea with tapioca pearl was produced by Guangdong Strong Group Co., Ltd (Guangdong, China). Each packet was dissolved in 120 ml boiling water, was then cooled to 20°C, and poured into a drinking bottle for mice. Animals were randomly assigned to two groups, including the bubble-tea group (*n* = 8) and the control group (*n* = 8). The bubble-tea group was given two drinking bottles-one filled with pure water and another with bubble tea. The bottles were renewed every day. The control-group mice were given two drinking bottles filled only with pure water.

### Body weight measurement

**FIGURE 1:**
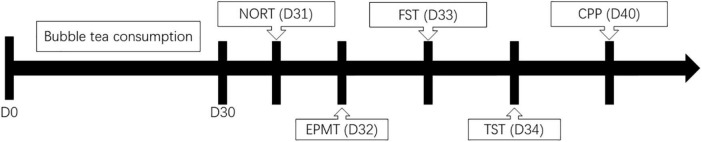
Study protocol.

The body weight of all mice in each study group was measured once every day for 30 days (Figure 1). Their measurements were taken at 8:00 am throughout the study using a digital balance (JA11002, Jinhua Instruments, Shanghai, China). The change in their body weight was calculated as the body weight measured on a given day, minus the body weight measured on Day 1.

### Novel object recognition test

Novel object recognition test was used to evaluate the short-term memory. The experiments were carried out in an open field box with black floor and wall (measuring 50 cm × 50 cm × 50 cm). Prior to the test, the mice were acclimatized to the test environment for 10 min per day for two consecutive days with no objects present. On the test day, the mice were placed in the test box, and after a 5-min habituation period, two objects were introduced in two corners (approximately 30 cm apart from each other). The objects used in this study were plastic blocks of the same size but different colors (measuring 9 cm × 5 cm × 9 cm). The time spent exploring each object was recorded during the subsequent 5 min period (defined as the training session). Then the mice were returned to their home cage. After a 120-min rest period, the mice were placed in the test chamber again, in which one of the familiar objects used in the previous training session was replaced with a new object. Since mice showed no preference for particular objects or locations, the one-side objects were regularly replaced. The time spent exploring each object was recorded during the subsequent 5 min period (defined as the test session). The animals were regarded to be exploring when they were facing, sniffing, or biting the object. To prevent the accumulation of olfactory cues, the boxes and objects were cleaned between trials using 75% ethanol. The recognition index, which measures memory preference, was measured as the proportion of time spent exploring the novel object over total exploration time, and it reflected the short-term memory ability. Experimenters were blind to treatments. The NORT instrument was purchased from RWD Life Science Co., Ltd. (Shenzhen, Guangdong, China).

### Elevated plus maze test

Elevated plus maze test is an experimental method to measure the anxiety response of animals, using their exploratory nature to novel environments and their fear of high open arms to form conflicting behaviors to examine their anxiety state. EMPT instrument consists of two open arms (25 cm × 5 cm) and two enclosed arms of the same size at opposite sides. The closed arm is surrounded by a 15 cm high wall, while the open arm is surrounded by a low wall of 3 mm. The part of the two arms that overlap is the central square area (5 cm × 5 cm). Both arms are 55 cm above the floor. Each mouse was placed into the instrument from the same position in the central square area. The entire apparatus was cleaned using 75% ethanol for the test of each subject. After each mouse was placed in the central square area, a dedicated camera was used to record the trajectory over the subsequent 5 min. The number of entries and the time spent were recorded for each arm. The number of entries and the time spent in the open arm reflected the general behavior of the mice. The less common entrance of the mice into the open arms of the maze, as well as the decreased amount of time they spent, was considered anxiety-like behavior. The EPMT instrument was purchased from RWD Life Science Co., Ltd. (Shenzhen, Guangdong, China).

### Forced swim test

Forced swim test was carried out as a highly reliable test for evaluating depressive-like behavioral state. The mice were forced to swim individually in a transparent plexiglass cylindrical tank (30 cm × 20 cm) containing water maintained at 22 ± 2°C. The water level was adjusted to a height of 15 cm. After an initial period of vigorous activity, the animals assume a type of immobile posture. A mouse is said to be immobile when it ceases struggling and makes the minimal movement of limbs to keep the head above water. The total duration of the test was six min with the immobility time recorded. The FST instrument was purchased from RWD Life Science Co., Ltd. (Shenzhen, Guangdong, China).

### Tail suspension test

Similar to FST, the immobility time of TST reflects a state of “behavioral despair” in depression. A small piece of medical adhesive tape was placed approximately 1–1.5 cm from the tip of the tail and the mice were hung individually for a period of 5 min at 15 cm away from the nearest surface. The climbing of the tails was prevented by the application of plastic tubing around them prior to the use of the adhesive tape. The duration of immobility was then measured for 10 min of the test. The TST instrument was purchased from RWD Life Science Co., Ltd. (Shenzhen, Guangdong, China).

### Conditioned place preference

Conditioned place preference was used to measure the rewarding effects of drugs on experimental animals. The CPP score reflects the learning association of animals to environmental cues and stimuli of interest. The test mouse was allowed to move freely between transparent and black boxes for a 15 min session once a day, for 3 days as preconditioning. On the third day, the time spent in each box was measured. There was no significant difference between time spent in the black compartment with a smooth floor and the white compartment with a textured floor, indicating that there was no place preference before conditioning. On days 4, 6, and 8, the mice were given bubble tea and confined to either the transparent or black box for 30 min. On days 5, 7, and 9, mice were given pure water and placed in the opposite conditioning box for 30 min. On day 10, the post-conditioning test was performed without bubble tea or pure water, and the time spent in each box was measured for 15 min. A counterbalanced protocol was used to nullify any initial preference by the mouse. The CPP score was designated as the time spent in the bubble tea-conditioning sites, minus the time spent in the water-conditioning sites. The CPP instrument was purchased from RWD Life Science Co., Ltd. (Shenzhen, Guangdong, China).

### Statistical analysis

Each behavior test was recorded and analyzed using the video-tracking option SMART 3.0 (Panlab Harvard Apparatus, Spain). All statistical analyses were performed using SPSS 16.0 (SPSS, Chicago, IL, USA). GraphPad Prism 5 software (GraphPad Software Inc., La Jolla, CA, USA) was used to draw figures. Results are expressed as means ± S.E.M. Comparison between experimental and control groups was performed by independent-samples *t*-test. The change in the body weight was analyzed using a repeated-measures analysis of variance. A *P*-value of < 0.05 was considered to be statistically significant.

## Results

### Effect of bubble tea administration on body weight

**FIGURE 2:**
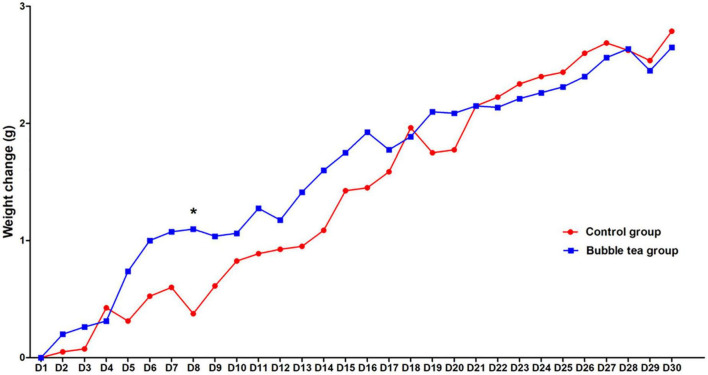
Effect of bubble tea administration on body weight. *P < 0.05.

The weight gain was recorded at the beginning of bubble tea treatment, and per day during the 30 days. There was a significant time effect on weight change of the two groups (*F*_time_ = 36.83, *P* < 0.01, Figure 2). Interestingly, we did not find a significant group effect or interaction (time × group) on the weight change in the two groups (*F*_group_ = 0.18, *P* = 0.68; *F*_time × group_ = 1.08, *P* = 0.37). Mice in either the bubble-tea group or the control group gained weight over time. The body weight of the bubble-tea group increased faster than that of the mice in the control group from the first day to the eighth day. *Post-hoc* analysis showed a significant difference between the bubble tea group and the control group at Day 8 (0.38 ± 0.64 vs. 1.10 ± 0.74, *t* = −2.88, *P* < 0.05). The body weight of the mice in the bubble-tea group did not change significantly compared with that of the mice control group on any other day of measurement (Figure 2).

### Effect of bubble tea administration on cognition function

**FIGURE 3:**
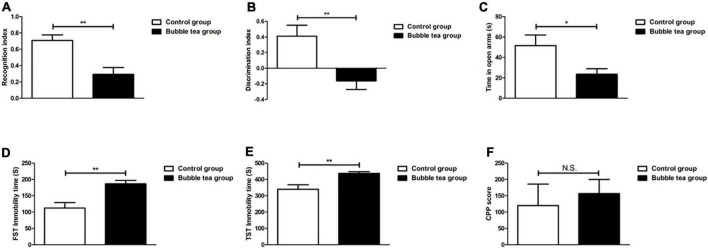
Effect of bubble tea administration on cognition function, anxiety and depressive, and addiction behavior. **(A)** Columns represent the recognition index in NORT. Independent-samples t-test, two tailed. **(B)** Columns represent the discrimination index in NORT. Independent-samples t-test, two tailed. **(C)** Columns represent the time that mice were in the open arm of the EPMT. Independent-samples *t*-test, two tailed. **(D)** Columns represent the immobility time of mice in the FST. Independent-samples *t*-test, two tailed. **(E)** Columns represent the immobility time of mice in the TST. Independent-samples *t*-test, two tailed. **(F)** Columns represent the CPP scores of mice. Independent-samples *t*-test, two tailed. *P < 0.05 and **P < 0.01.

In the NORT, mice that received pure water or bubble tea showed significant differences in the recognition index (0.71 ± 0.20 vs. 0.29 ± 0.23, *t* = 3.83, *P* < 0.01) (Figure 3A). Mice in the bubble-tea group did not like to spend more time in front of the new object. We also measured the discrimination index (0.41 ± 0.40 vs. −0.16 ± 0.31, *t* = 3.19, *P* < 0.01) (Figure 3B).

### Effect of bubble tea administration on anxiety and depressive behavior

Anxiety was tested using the EPMT. The mice in the control group showed less anxiety-like behavior by spending more time in the open arms than those in the bubble tea group during the test. On the contrary, the bubble tea-treated mice spent significantly less time in the open arms, indicating an increase in anxiety (*t* = 2.39, *P* = 0.03, Figure 3C). In FST, bubble tea treatment produced a significantly increased immobility time (186.58 ± 29.67 s) as compared to pure water treated group (112.50 ± 46.67 s) (*t* = −3.79, *P* < 0.01) (Figure 3D). Similarly, the immobility time in the TST was also significantly increased by bubble tea treatment (437.63 ± 27.72 s) compared to the treatment with pure water (340.24 ± 77.22 s) (*t* = −3.36, *P* < 0.01) (Figure 3E).

### Effect of bubble tea administration on addiction behavior

We investigated the rewarding effects of bubble tea, using the CPP paradigm, which measures the rewarding properties of abused drugs. Independent-samples *t*-test revealed no significant difference between the two groups (*t* = −0.47, *P* = 0.65) (Figure 3F).

## Discussion

To our knowledge, this is the first study investigating the effects of bubble tea administration on addictive, anxiety, and depressive behavior in a controlled setting using animal models. The main findings of this study are as follows. First, long-term bubble tea administration did not induce addictive behavior. Second, chronic bubble tea use has a negative effect on emotion. Mice in the bubble tea group showed anxiety and depressive-like behavior compared with those in the control group. Third, long-term administration of bubble tea resulted in cognitive decline in experimental mice. Forth, drinking bubble tea for a long time has little effect on the weight gain of mice, but there will be a period of rapid weight increase in the early stage.

In spite of the potential benefits of bubble tea, concerns over their safety have been raised. The first concern of bubble tea consumers is whether drinking it for a long time will lead to weight gain. Bubble tea is a kind of SSB. Malik’s systematic review and meta-analysis of prospective cohort studies and RCTs provide evidence that SSB consumption promotes weight gain in children and adults (11). Ritze’ study also measured the weight change of mice after 8 weeks 30% sucrose diet (12). Sucrose group mice gained 2.3 times more weight than the control mice at the end of 8 weeks. Lee and colleagues compared weight changes of mice with carbonated soda, sweetened milk coffee, and chocolate-added cocoa treatment (13). Compared with the group treated with water, the SSB-treated group with chocolate-added cocoa or sweetened milk coffee showed greater increases in body weight.

Different from other studies, Lieder and colleagues changed both the diet and water of mice to detect risk factors for weight change (14). Some mice received normal chow. Other mice received conventional cheeseburgers as a typical Western-type diet. Water also dived into tap water and caffeinated sugar-sweetened soft drink as the prototypical SSB. Unexpectedly, although the overall energy intake was higher in the cheeseburger groups, there was no difference in body weight between the SSB and tap water groups after 24 weeks. Our study found no significant difference in weight change between the bubble-tea group and the control group, either. However, there was a time effect both in the two groups. Following the time change, mice from the bubble-tea or the control group gained weight over time. We speculated that the lack of difference in weight gain between the two groups might be related to the mood changes of the mice. Notably, there was a difference between the two groups on the eighth day, perhaps because the mice of the bubble-tea group got emotional change. It is worth noting that we did not conduct behavioral testing on the eighth day, and this difference may also be caused by other reasons. We will further explore the hypothesis in future studies.

The second question is addiction. Most bubble tea recipes contain a tea base that is mixed with fruit juice or milk, and chewy tapioca starch balls or fruit jellies are often added. Tea is probably the only potentially addictive ingredient above-mentioned. However, a potentially addictive factor of sugar cannot be ignored. An increasingly popular theory is that sugar acts as an addictive agent, eliciting neurobiological changes similar to those seen in drug addiction (15–18). Westwater’s review showed that there is little evidence to support sugar addiction in humans, and findings from the animal literature suggest that addiction-like behaviors, such as bingeing, occur only in the context of intermittent access to sugar (15). Consist with this result, we also found that bubble tea cannot induce addiction. This negative result may be related to the choice of ingredients in bubble tea. The ingredients in bubble tea powder we selected include granulated sugar, vegetable fat (glucose syrup, hydrogenated coconut oil, whey powder, sodium caseinate, diglycerol fatty acid ester, stearyl lactate, sodium dipotassium phosphate, sodium tripolyphosphate, and silicon dioxide), milk powder (skim milk powder and whole milk powder), instant black tea powder, and edible essence. These ingredients lack substances that can lead to addictive behavior.

Tea drinking originated from China around 4,000–5,000 years ago and was spread to the rest of the world (19). Today, bubble tea has also spread from China to the world, becoming a very popular beverage worldwide (20, 21). Previous findings show that all major tea types can function through multiple pathways to collectively reduce the risk of depression (22–24). The antidepressant materials include predominantly L-theanine, polyphenols, and polyphenol metabolites (25). Bubble tea just has a little instant black tea powder. Maybe, that is the reason why chronic bubble tea use has no antidepressant effect. Conversely, mice in the bubble-tea group showed anxiety and depressive-like behavior compared with the mice in the control group. The exact mechanism needs to be further studied. In general, current evidence suggests that both green tea and black/oolong tea have beneficial cognitive effects that are reversed when sugar, milk, and other ingredients were added (26–28). In our study, the long-term bubble-tea administrated mice showed a lower recognition index than the mice in the control group. The recognition index reflects the cognitive function of the mice. Unfortunately, the exact mechanism of why chronic bubble tea administration causes cognitive decline in mice is still unclear. We speculate that sugar is probably the most important factor. Based on Kendig’s review, sugar can impair spatial learning and memory (29).

This study has some limitations. First, we did not weigh the chow of each mouse daily in our study. As in Lieder’s study, the mice that received SSB water may reduce their chow intake. Second, we did not measure biomarkers in the two groups. Third, we did not measure the daily amount of bubble tea consumed by the animals.

## Conclusion

We showed that long-term administration of bubble tea could not induce addictive behavior in mice. Meanwhile, the long-term effects of bubble tea on weight were also very limited. However, long-term consumption of bubble tea can lead to anxiety and depression-like behaviors and impair cognitive function in mice. This is a preclinical study and the results obtained need to be further confirmed by more rigorous double-blind, randomized-control clinical studies.

## Data availability statement

The raw data supporting the conclusions of this article will be made available by the authors, without undue reservation.

## Ethics statement

The animal study was reviewed and approved by the Institutional Animal Ethics Committee of Chaohu Hospital of Anhui Medical University.

## Author contributions

KZ wrote the main manuscript text. YiY prepared the figures. YaY, SS, YiY, BL, ML, XY, and LZ performed the study and collected the data. All authors reviewed the manuscript.
